# Engineering Novel Amphiphilic Platinum(IV) Complexes to Co-Deliver Cisplatin and Doxorubicin

**DOI:** 10.3390/molecules29174095

**Published:** 2024-08-29

**Authors:** Wjdan Jogadi, Man B. Kshetri, Suha Alqarni, Arpit Sharma, May Cheline, Md Al Amin, Cynthia Sheets, Angele Nsoure-Engohang, Yao-Rong Zheng

**Affiliations:** 1 Department of Chemistry and Biochemistry, Kent State University, 236 Integrated Sciences Building, Kent, OH 44242, USAmkshetri@kent.edu (M.B.K.); salqarn3@kent.edu (S.A.); malamin1@kent.edu (M.A.A.);; 2 Department of Chemistry, University of Bisha, Bisha 67714, Saudi Arabia

**Keywords:** platinum(IV) prodrugs, cisplatin, doxorubicin, combination therapy

## Abstract

In this study, we report a novel platinum–doxorubicin conjugate that demonstrates superior therapeutic indices to cisplatin, doxorubicin, or their combination, which are commonly used in cancer treatment. This new molecular structure (**1**) was formed by conjugating an amphiphilic Pt(IV) prodrug of cisplatin with doxorubicin. Due to its amphiphilic nature, the Pt(IV)–doxorubicin conjugate effectively penetrates cell membranes, delivering both cisplatin and doxorubicin payloads intracellularly. The intracellular accumulation of these payloads was assessed using graphite furnace atomic absorption spectrometry and fluorescence imaging. Since the therapeutic effects of cisplatin and doxorubicin stem from their ability to target nuclear DNA, we hypothesized that the amphiphilic Pt(IV)–doxorubicin conjugate (**1**) would effectively induce nuclear DNA damage toward killing cancer cells. To test this hypothesis, we used flow the cytometric analysis of phosphorylated H2AX (γH2AX), a biomarker of nuclear DNA damage. The Pt(IV)–doxorubicin conjugate (**1**) markedly induced γH2AX in treated MDA-MB-231 breast cancer cells, showing higher levels than cells treated with either cisplatin or doxorubicin alone. Furthermore, MTT cell viability assays revealed that the enhanced DNA-damaging capability of complex **1** resulted in superior cytotoxicity and selectivity against human cancer cells compared to cisplatin, doxorubicin, or their combination. Overall, the development of this amphiphilic Pt(IV)–doxorubicin conjugate represents a new form of combination therapy with improved therapeutic efficacy.

## 1. Introduction

Platinum-based drugs, e.g., cisplatin, carboplatin, and oxaliplatin, are widely used as anticancer agents in the treatment of various tumors, including testicular, lung, ovarian, breast, and colon cancer [1,2]. Their mechanism of action involves the formation of platinum–DNA adducts, leading to apoptosis in cancer cells [1,3,4]. The effectiveness of platinum-based therapies is often enhanced through their combination with other chemotherapeutic agents, targeted therapies, and immunotherapies. This synergistic approach, known as combination therapy, can overcome resistance mechanisms, improve drug delivery, and enhance efficiency [5,6,7]. In combination therapy, the strategy involves the simultaneous administration of multiple chemotherapeutic agents that target different cellular pathways, reducing drug resistance and enhancing therapeutic efficacy [8,9,10,11,12]. For example, platinum drugs (cisplatin or carboplatin) are commonly combined with drugs like doxorubicin, an anthracycline chemotherapeutic agent known for its noncovalent intercalation into DNA, thereby triggering DNA damage [13,14,15,16,17,18,19,20]. One of the significant challenges in combination drug therapy arises from the varying pharmacokinetic properties of different drugs. These properties include absorption, distribution, metabolism, and excretion; these might vary substantially between drugs. As a result, each drug in a combination regimen may be distributed differently within the body, leading to varying concentrations, timings of administration, and actions at the target sites [10,11]. This differential distribution complicates the control of the accumulation of each specific drug, making it challenging to ensure that each drug reaches its target site in the optimal concentration for maximum efficacy. Also, this leads to increased toxicity. There is a strong demand for new approaches for coordinating the delivery of multiple drugs to the same site of action, where achieving synergistic effects without causing adverse interactions or toxicity can be complex [1,2].

Amphiphilic Pt(IV) complexes are distinctive Pt(IV) complexes featuring a hydrophobic tail and a hydrophilic head group that are capable of releasing axial ligands and Pt(II) payloads upon intracellular reduction. They have emerged as a promising new class of Pt-based anticancer agents, leveraging innovative drug delivery strategies and cancer biology to overcome the limitations of traditional Pt(II) drugs [10,14,21,22,23,24,25]. Designed to mimic the structure of fatty acids, fatty acid-like amphiphilic Pt(IV) prodrugs of cisplatin engage in noncovalent interactions with human serum albumin and CD36 to facilitate efficient Pt drug delivery [21,22,26,27]. These Pt complexes exhibit remarkable stability in whole human blood, reducing their rate of reduction by reducing agents, and possess unique cancer cell targeting capabilities and mechanisms of action [23,28]. Such amphiphilic Pt(IV) prodrugs have demonstrated potent in vitro activity against a wide range of cancer types and promising in vivo efficacy in various mouse models. Importantly, amphiphilic Pt(IV) prodrugs can be chemically modified to adjust their biological activities and chemical properties [14,25,29,30,31,32,33]. Recent studies have shown the potential of incorporating these novel Pt(IV) prodrugs into nanoparticles for drug delivery through either noncovalent encapsulation or covalent conjugation based on their amphiphilic structures [14]. These Pt complexes resemble innovative lipid–prodrug molecules, which exhibit significant absorption, distribution, metabolism, excretion, and toxicity (ADMET) advantages and can potentially reduce systemic toxicity by selectively releasing the active drug at the target site [34,35,36,37,38,39]. Overall, amphiphilic Pt(IV) prodrugs represent a highly diverse and unique Pt scaffold with promising mechanisms of action, potentially serving as a powerful tool for developing new cancer therapies.

**Figure 1:**
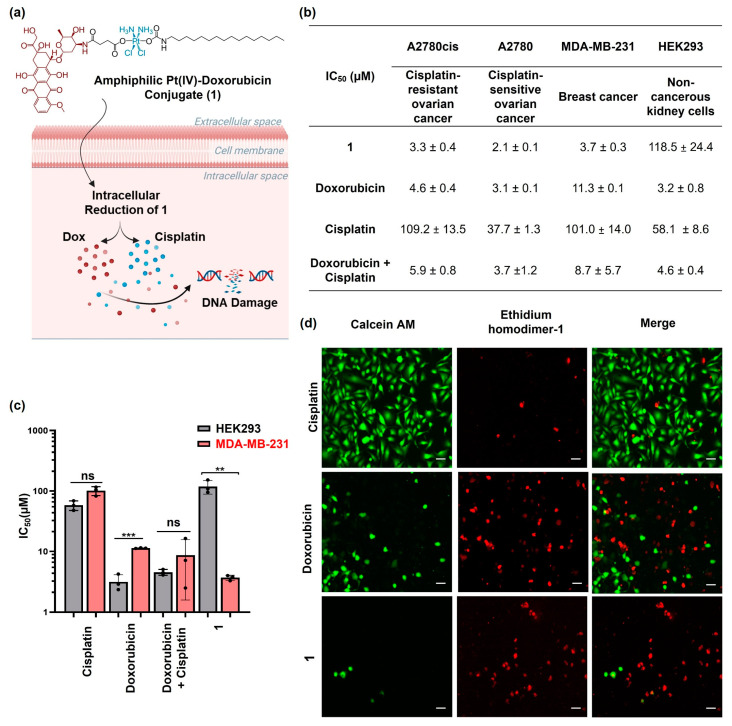
(**a**) The structure of amphiphilic Pt(IV)–doxorubicin conjugate (**1**) and its proposed mechanism of action (created with BioRender.com, accessed on 16 July 2024); (**b**) IC_50_ values determined by MTT assays for **1**, doxorubicin, cisplatin, and their combination against A2780cis, A2780, MDA-MB-231, and HEK293 cell lines; (**c**) bar graph depicting the IC_50_ values of **1** in comparison with those of doxorubicin, cisplatin, and their combination in a 1:1 ratio. (**d**) Live/dead assay images of MDA-MB-231 cells treated with cisplatin, doxorubicin, and **1** (10 µM) for 24 h. Scale bar = 100 µm. ** *p* < 0.01 and *** *p* < 0.001 per *t* test (*n* = 3, mean ± SEM), ns: no significance.

In this paper, we report a novel amphiphilic Pt(IV)–doxorubicin conjugate (**1** shown in Figure 1a) as a combined-drug scaffold. This new molecular scaffold (**1**) was formed by conjugating an amphiphilic Pt(IV) prodrug of cisplatin with doxorubicin, enabling the efficient delivery of both payloads to target cancer cells and having superior therapeutic indices to cisplatin and doxorubicin.

## 2. Results and Discussion

### 2.1. Synthesis and Characterization of the Amphiphilic Pt(IV)–Doxorubicin Conjugate (**1**)

As shown in Figure S1 in the supporting Information, the amphiphilic Pt(IV)–doxorubicin conjugate (**1**) was synthesized using doxorubicin and the previously reported precursor, C16Pt [21]. In brief, doxorubicin was conjugated to C16Pt through an amide bond formation catalyzed by HATU, resulting in an overall yield of 50.5%. The amphiphilic Pt(IV)–doxorubicin conjugate (**1**) was thoroughly characterized by ^1^H and ^13^C NMR spectroscopy, HPLC, and electrospray ionization mass spectrometry (ESI-MS). In the ^1^H NMR spectrum (Figure S2), the broad signal at 6.58 ppm corresponds to the amine groups of the Pt(IV) center, while the signal at 6.51 ppm is attributed to the carbamate group. The signal at 2.86 ppm is assigned to the CH_2_ group adjacent to the carbamate, and the terminal CH_3_ at the C16 tail is observed at 0.86 ppm. The signal at 7.94 ppm is attributed to the aromatic protons of doxorubicin. HPLC analysis (Figure S4) of the final product indicated a purity of 97%. The ESI-MS spectrum (Figure S5) shows an isotopically resolved signal at *m*/*z* = 1226.4041, which is consistent with the theoretical value for compound **1** (*m*/*z* = 1226.4045).

### 2.2. Cytotoxicity Profiles of Amphiphilic Pt(IV)–Doxorubicin Conjugate (**1**)

The amphiphilic Pt(IV)–doxorubicin conjugate (**1**) is proposed as undergoing intracellular reduction (Figure 1a), releasing cisplatin and the doxorubicin payload (Dox) to induce DNA damage and eliminate cancer cells. The therapeutic effect of compound **1** was examined in vitro using a 3-(4,5-dimethylthiazol-2-yl)-2,5-diphenyltetrazolium bromide (MTT) assay and a live/dead cell imaging assay. We engaged a panel of human cancer cell lines of ovarian cancer (A2780cis and A2780) and breast cancer (MDA-MB-231) for assessing the in vitro efficacy and the noncancerous human kidney cell line (HEK293) for evaluating their selectivity. Compared to A2780 cells, the A2780cis cell line is a cisplatin-resistant ovarian cancer model used to evaluate the efficacy of novel chemotherapeutic agents that can overcome platinum resistance. The cells were treated with **1**, doxorubicin, cisplatin, or the combination of doxorubicin and cisplatin for 24 h. Then, the cell viability was assessed with MTT via the IC_50_ value, representing the drug concentration that was needed to inhibit 50% of cell viability relative to the control. As shown in Figure 1b and Figure S6, **1** exhibited higher cytotoxicity than doxorubicin and cisplatin or their combination across all the different cancer cell lines tested. For example, the IC_50_ value of **1** was 3.7 ± 0.2 μM (Figure 1c) against MDA-MB-231 breast cancer cells, lower than that of doxorubicin (IC_50_ = 11.3 ± 0.1 μM) and cisplatin (IC_50_ = 101.0 ± 14.0 μM) or their combination (IC_50_ = 8.7 ± 5.6 μM). Notably, the amphiphilic Pt(IV)–doxorubicin conjugate (**1**) did not exhibit significant cytotoxicity against noncancerous HEK293 cells, but both cisplatin and doxorubicin as well as their combination were highly toxic against noncancerous HEK293 cells (Figure 1c). In addition, we used a live/dead imaging assay to further validate the anticancer activity of complex **1**. This involved the use of ethidium homodimer-1 with calcein AM (Figure 1d). The live cells were identified by the observation of green fluorescence after staining with calcein AM, while the dead cells exhibited a red signal upon interaction with the MDA-MB-231 cells treated with complex **1**, having a much smaller population of live cells compared to those treated with cisplatin or doxorubicin. This observation is consistent with the results of the MTT assay. Overall, the combined evidence from the MTT assays and live/dead cell imaging assays indicates that the amphiphilic Pt(IV)–doxorubicin conjugate (**1**) is a highly potent anticancer agent. Its efficacy surpasses that of both cisplatin and doxorubicin, individually or combined, against human cancer cells while exhibiting minimal toxicity to noncancerous human cells.

### 2.3. Cellular Accumulation of the Amphiphilic Pt(IV)–Doxorubicin Conjugate (**1**)

**Figure 2:**
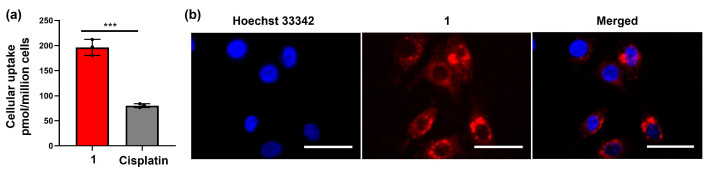
(**a**) Cellular accumulation of **1** ([Pt] = 2 µM) and cisplatin ([Pt] = 20 µM) in MDA-MB-231 cells; (**b**) images of MDA-MB-231 cells treated with **1** (2 μM, 24 h) and labeled with Hoechst for 3 h at 37 °C, 5% CO_2_. Scale bar = 100 µm. *** *p* < 0.001 by *t* test (*n* = 3, mean ± SEM).

To better understand the superior therapeutic effects of the amphiphilic Pt(IV)–doxorubicin conjugate (**1**), we investigated its mechanism of action. Cell entry is a key step in this process. We utilized graphite furnace atomic absorption spectrometry (GFAAS) to analyze the intracellular Pt contents and live cell fluorescence imaging to validate the intracellular doxorubicin payloads. In the GFAAS experiment, MDA-MB-231 cells were treated with **1** ([Pt] = 2 µM) and cisplatin ([Pt] = 20 µM). After 24 h, the cellular Pt contents were measured. The results (Figure 2a) show that the cellular accumulation of cisplatin was only 80.2 ± 3.8 pmol Pt/10^6^ cells, while the accumulation of **1** was significantly higher at 196.5 ± 16.1 pmol Pt/10^6^ cells, 2.4 times greater. The results indicate that the amphiphilic Pt(IV)–doxorubicin conjugate (**1**) facilitates the cell entry of cisplatin payloads. In the imaging experiment, the fluorescence signal of doxorubicin was used to track the intracellular accumulation and localization of the doxorubicin payloads after treatment with **1** (Figure 2b). MDA-MB-231 cells were treated with **1** for 24 h and then subsequently stained with the nuclear dye Hoechst 33342. The images (Figure 2b) show that **1** effectively delivered doxorubicin into cancer cells, with most of the payload localized in the cytoplasm and some in the nucleus. Collectively, the combined evidence from GFAAS and cellular imaging demonstrates that the amphiphilic Pt(IV)–doxorubicin conjugate (**1**) effectively delivers both cisplatin and doxorubicin across the cellular membrane, leading to the significant intracellular accumulation of both payloads.

### 2.4. Cellular Responses of the Amphiphilic Pt(IV)–Doxorubicin Conjugate (**1**)

**Figure 3:**
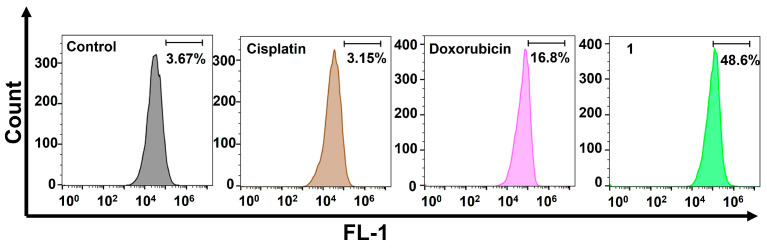
Flow cytometric analysis of nuclear DNA damage using the γH2AX biomarker in MDA-MB-231 cells treated with cisplatin (10 µM), doxorubicin (1 µM), and the amphiphilic Pt(IV)–doxorubicin conjugate (**1**, 1 µM) for 24 h.

We then examined the DNA damage caused by the amphiphilic Pt(IV)–doxorubicin conjugate (**1**). Cisplatin works by forming covalent DNA adducts that disrupt DNA replication and transcription, leading to apoptosis. Doxorubicin, on the other hand, noncovalently intercalates into DNA strands and inhibits topoisomerase II, causing DNA breaks and cell death. Both agents are well known for their effectiveness in inducing DNA damage. γH2AX, a phosphorylated form of H2AX, is a biomarker of nuclear DNA damage. In our experiment, we treated MDA-MB-231 cells with cisplatin, doxorubicin, or **1**, and then we performed flow cytometric analysis to detect γH2AX levels. As shown in Figure 3, the cells treated with **1** exhibited significantly higher levels of γH2AX than those treated with cisplatin or doxorubicin. Specifically, the γH2AX levels in **1**-treated cells were 15.4 times higher than in cisplatin-treated cells and 2.89 times higher than in doxorubicin-treated cells. These substantial increases indicate that **1** induces more extensive nuclear DNA damage. These findings are consistent with the previously mentioned results from the MTT assay, live/dead imaging study, and cellular accumulation experiment.

**Figure 4:**
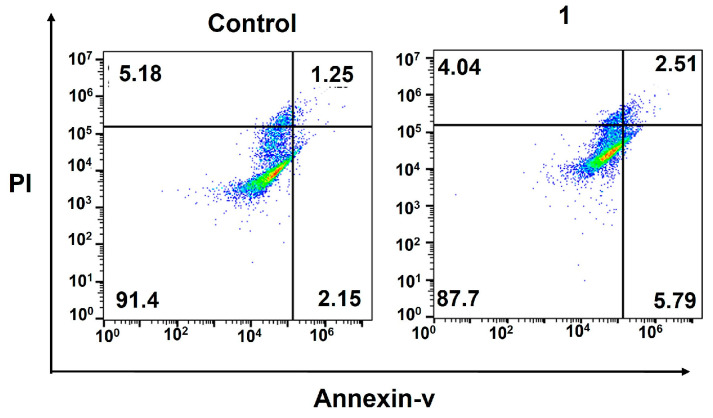
Flow cytometric analysis of apoptosis using the Annexin V/PI biomarkers in MDA-MB-231 cells treated with the amphiphilic Pt(IV)–doxorubicin conjugate (**1**, 0.5 µM) for 72 h.

Finally, we confirmed the cell death induced by DNA damage using a flow cytometric analysis of apoptosis. During early apoptosis, phosphatidylserine, normally located on the inner lipid layer, moves to the outer layer. FITC–annexin V, a green fluorescent dye-conjugated antibody, selectively binds to phosphatidylserine on the outer membrane, enabling the flow cytometric analysis of apoptosis. In late apoptosis, cell membranes become damaged, allowing propidium iodide (PI) dye to penetrate and produce a red signal in flow cytometry. The occurrence of apoptosis in **1**-treated MDA-MB-231 cells was assessed using a dual-staining annexin V/PI flow cytometric assay. The results (Figure 4) clearly indicate that the amphiphilic Pt(IV)–doxorubicin conjugate (**1**) effectively induces apoptosis in triple-negative breast cancer cells. After incubating cells with **1** ([Pt] = 0.5 µM) for 72 h, a significant population of cells were in late (5.79%) and early (2.51%) apoptosis stages compared to those in the control sample, which only showed 1.25% and 2.15% of cells in late and early apoptosis, respectively.

Overall, the combined evidence suggests that the amphiphilic Pt(IV)–doxorubicin conjugate (**1**) not only enhances cellular accumulation of therapeutic agents but also induces significantly more DNA damage, leading to superior therapeutic effects compared to cisplatin or doxorubicin alone.

## 3. Conclusions

In this study, we developed and evaluated a novel amphiphilic Pt(IV)–doxorubicin conjugate (**1**) designed to enhance anticancer activity. Our findings showed that this conjugate significantly improves cellular uptake, intracellular accumulation, and cytotoxicity compared to cisplatin and doxorubicin alone. The Pt(IV)–doxorubicin conjugate demonstrated superior cytotoxicity against various human cancer cell lines, while showing minimal toxicity towards non-cancerous human kidney cells. This selectivity highlights its potential as a more effective and safer cancer therapy. Our mechanistic studies revealed that the conjugate facilitates the entry of both cisplatin and doxorubicin payloads into cells, resulting in substantial DNA damage, as evidenced by elevated γH2AX levels. This increased DNA damage led to higher apoptosis rates, as confirmed by flow cytometric analysis. The combined results from cytotoxicity assays, cellular accumulation studies, DNA damage analysis, and apoptosis evaluation indicate that the amphiphilic Pt(IV)–doxorubicin conjugate (**1**) outperforms traditional cisplatin and doxorubicin treatments. This novel conjugate holds promise for enhancing combination chemotherapy effectiveness, offering a new avenue for developing potent anticancer therapies with reduced side effects.

## 4. Materials and Methods

All reagents were purchased from Strem Chemicals (Newburyport, MA, USA), Sigma-Aldrich (St. Louis, MO, USA), or Alfa Aesar (Haverhill, MA, USA) and used without further purification. All reactions were carried out under normal atmospheric conditions. C16Pt was synthesized following a previously reported procedure [21]. A Bruker 400 NMR spectrometer (Bruker Corporation, Billerica, MA, USA) was used for NMR data acquisition (frequency: 400 MHz for ^1^H NMR; 100 MHz for ^13^C NMR). Chemical shifts in ^1^H and ^13^C{^1^H} NMR spectra were internally referenced to solvent signals (^1^H NMR: DMSO-d_6_ at δ = 2.50 ppm; ^13^C NMR: DMSO-d_6_ at δ = 40.45 ppm). For **1**, high-resolution mass spectra were recorded on an Exactive Plus mass spectrometer (Thermo Scientific, Bremen, Germany). Analytical HPLC was conducted on an Agilent 1100 system using C18 reverse-phase columns (Hypersil GOLD, 100 mm × 3 mm, 5 µm). GFAAS measurements were taken with a PerkinElmer PinAAcle 900Z spectrometer. Images were processed and intensities were quantified with ImageJ software (version 1.4.22, National Institutes of Health, Bethesda, MD, USA). The live/dead cell assay was carried out using an Invitrogen (Thermo Fisher Scientific, Waltham, MA, USA) LIVE/DEAD^TM^ Cell Viability Kit (Cat. No. L3224). Flowcytometry was carried out on a BD Bioscience Accuri C6 flow cytometer (BD Biosciences, San Jose, CA, USA).

Synthesis of the amphiphilic Pt(IV)–doxorubicin conjugate (**1**): C16Pt (108.9 mg, 0.150 mmol) [40], doxorubicin hydrochloride (90.0 mg, 0.155 mmol) and HATU (58.9 mg, 0.150 mmol) were combined in a 20 mL vial, then anhydrous DMF (2 mL) was added under inert gas and stirred at r.t. for 20 min. Then, DIPEA (108 µL, 0.620 mmol) was added dropwise. The reaction mixture was stirred in the dark at r.t. for 16 h. The reaction mixture was then centrifuged, and the supernatant was added into a brine solution (3 mL). Then, the precipitate was isolated by centrifuging, followed by decanting the supernatant. The solid was subsequently washed with water twice (1 mL) and lyophilized for 16 h. The product was purified via flash column chromatography on silica gel (eluent MeOH/DCM 1% to 10%). The excess solvent was removed by rotavap, and then the solid was dried in a vacuum. The yield was 50.5%. HR-MS (positive mode) for [C_48_H_73_Cl_2_N_4_O_16_Pt]^+^: *m*/*z* calc: 1226.4045, obsd: 1226.4041. Purity: 97% determined by HPLC.

^1^H NMR (400 MHz, DMSO-d_6_): δ: 0.856 (NHCH_2_(CH_2_)_14_C*H*_3_, t, 3H, *J* = 6.8 Hz), 1.125 (doxorubicin, OCH(C*H*_3_)CH(OH)CH(NHCO)CH_2_, d, 3H, *J* = 6.4 Hz), 1.238 (NHCH_2_(C*H*_2_)_14_CH_3_, s, 28H), 1.850 (doxorubicin, CH(NHCO)*CH_2_*CH(OCH)OCH, td, 2H, *J* = 13.2, 4.0 Hz), 2.166 (doxorubicin, OCH(Ph)C*H*_2_C((CO)CH_2_)(OH)CH_2_, dd, 2H, J = 28.3 Hz), 2.326 (CO(C*H*_2_)_2_CO, dt, 4H, J = 55.8, 7.5 Hz), 2.859 (NHCH_2_(CH_2_)_14_CH_3_, q, 2H, J = 7.5 Hz), 2.992 (doxorubicin, CH_2_C((CO)CH_2_)(OH)CH, d, 2H, J = 4.6 Hz), 3.392 (doxorubicin, OCH(CH_3_)CH(OH)CH(NHCO)CH_2_, q, 1H, *J* = 5.0 Hz), 3.958 (doxorubicin, OCH(CH_3_)CH(OH)CH(NHCO)C*H*_2_Ph, quin, 1H, *J* = 8.2 Hz), 3.999 (doxorubicin, PhOC*H*_3_, s, 3H), 4.162 (doxorubicin, OC*H*(CH_3_)CH(OH)CH(NHCO)CH_2_, qd, 1H, *J* = 5.9, 1.3 Hz), 4.576 (doxorubicin, C(CO)C*H*_2_OH, d, 2H, *J* = 5.8 Hz), 4.749 (doxorubicin, OCH(CH_3_)CH(O*H*)CH(NHCO)CH_2_, d, 1H, J = 6.6 Hz), 4.872 (Doxorubicin, C(CO)CH_2_O*H*, t, 1H, *J* = 6.0 Hz), 4.960 (doxorubicin, PhC*H*(CH_2_)OCH, t, 1H, J = 4.6 Hz), 5.230 (doxorubicin, CHOC*H*(CH_2_)OCH(CH_3_)CH, d, 1H, *J* = 2.9 Hz), 5.487 (doxorubicin, (CH_2_)_2_C(O*H*)COCH_2_, s, 1H), 6.510 (N*H*CH_2_(CH_2_)_14_CH_3_, t, 1H, *J* = 6.0 Hz), 6.577 (N*H*_3_, m, 6H), 7.605 (CON*H*CH(CH_2_)CH(OH)CH, d, 1H, *J* = 7.9 Hz), 7.676 (doxorubicin, (CO)CH=CH-C*H*=C(OCH_3_), dd, 2H, *J* = 5.9, 3.9 Hz), 7.936 (doxorubicin, (CO)C*H*=C*H*-CH=C(OCH_3_), dd, 2H, *J* = 3.3, 2.6 Hz), 13.309 (doxorubicin, PhO*H*, s, 2H).

^13^C NMR (100 MHz, DMSO-d_6_): δ: 214.4, 187.0, 187.0, 171.2, 164.4, 161.3, 156.6, 155.1, 136.7, 136.0, 135.2, 134.6, 120.5, 120.2, 119.5, 111.3, 111.1, 100.9, 75.4, 70.2, 68.4, 67.1, 64.1, 57.1, 45.5, 37.1, 32.6, 31.9, 31.8, 30.3, 30.1, 29.5, 29.4, 29.2, 26.9, 22.6, 17.5, 14.4.

### 4.1. Cell Culture

A2780 and A2780cis cell lines were purchased from Sigma-Aldrich (St. Louis, MO, USA) and cultured with RPMI 1640 medium containing L-glutamine (Corning, NY, USA), supplemented with 10% FBS (Atlanta Biologicals, Flowery Branch, GA, USA) and 1% penicillin–streptomycin (Corning, NY, USA). The MDA-MB-231 and HEK293 cell lines were obtained from the American Type Culture Collection and cultured in DMEM 1 g/L glucose, with L-glutamine and sodium pyruvate (Corning), supplemented with 10% FBS and 1% penicillin–streptomycin (Corning, NY, USA). All cell lines were cultured at 37 °C under an atmosphere containing 5% CO_2_. Cells were passaged upon reaching 80–90% confluence via trypsinization and split into a ratio of 1:5.

### 4.2. MTT Assays

Cytotoxicity profiles of **1**, cisplatin, and doxorubicin against different cell lines (A2780, A2780cis, MDA-MB-231, HEK293) were evaluated by MTT assays. A 96-well plate was seeded with a volume of 100 μL of RPMI or DMEM containing 8 × 10^4^ cells/m overnight. The plates were incubated for 24 h at 37 °C, 5% CO_2_ to allow for adherence of cells. A specific volume of RPMI or DMEM (50 μL) at various concentrations of compound **1**, cisplatin, doxorubicin, or a 1:1 combination of both was added to each well of the microplates. A DMSO solution of compound **1** was used, with the DMSO concentration maintained below 0.5% in the assay. The C16 tail enables the Pt complex to solubilize in the cell culture medium through interaction with albumin [21]. After 24 h, MTT (30 μL, 5.0 mg/mL in PBS, Alfa Aesar, Haverhill, MA, USA) was added to each well of the microplates. After 3 to 4 h, the medium was aspirated, and DMSO (200 μL) was added to each well. The plates were shaken gently on a shaker at r.t. for 10 min. Subsequently, a BioTek ELx800 plate reader (BioTek Instruments, Winooski, VT, USA) was used to measure the absorbance of purple formazan at a wavelength of 562 nm. The IC_50_ values were calculated with the origin program. all experiments were conducted in triplicate with three independent experiments.

### 4.3. Live/Dead Cell Viability Assays

MDA-MB-231 cells were cultured in imaging dishes (MatTek, Ashland, MA, USA) at a concentration of 5 × 10^4^ cells with 2 mL of complete medium and were incubated for 24 h at 37 °C, 5% CO_2_. The cells were then treated with cisplatin, **1**, or doxorubicin (10 µM) and were then incubated for 24 h at 37 °C, at 5% CO_2_. For the experiment, the cells were rinsed with 1 mL of PBS. A 200 μL volume of the live/dead working solution, consisting of a mixture of 2 µM calcein AM and 2 μM in PBS, was added to the plates and then incubated at room temperature for 30 min. The recording of images was performed using an Olympus IX70 inverted epifluorescence microscope that was coupled to a digital CCD camera (QImaging). The images were analyzed, and the intensities were measured using ImageJ (version 1.4.22, National Institutes of Health, Bethesda, MD, USA). 

#### GFAAS Analysis of Cellular Pt Contents in MDA-MB-231 Cells

MDA-MB-231 cells were seeded in a 6-well plate at a concentration of 2 × 10^5^ cells per well and incubated at 37 °C, 5% CO_2_ overnight. Next day, the cells were treated with **1** ([Pt] = 2 μM) or cisplatin ([Pt] = 20 μM) for 24 h at 37 °C, 5% CO_2_. The primary goal was to demonstrate that complex **1** can accumulate in cells more effectively than cisplatin, even when a higher dosage of cisplatin is used. Based on pilot tests, 20 μM of cisplatin was chosen as it provided a measurable intracellular Pt concentration in GFAAS measurements without causing significant cell death. The surviving cells were collected using trypsinization and counted. The surviving cells were collected using trypsinization and counted. The cells were subsequently digested in 200 μL of 65% HNO_3_ at room temperature overnight. The platinum (Pt) concentrations in the cells were determined using GFAAS. The experiments were carried out triplicate.

### 4.4. Fluorescence Imaging

MDA-MB-231 cells were cultured in imaging dishes (MatTek) at a concentration of 6 × 10^4^ cells with 2 mL of complete medium incubated at 37 °C, 5% CO_2_ for 24 h. The next day, the cells were treated with **1** (2 µM), and then the cells were incubated for 24 h at 37 °C, 5% CO_2_. The next day, Hoechst dye was used to label the cells for 3 h. Then, the medium was taken out, the cells were washed with PBS, and the fluorescence was visualized with a confocal microscope using an Olympus IX70 inverted epifluorescence microscope equipped with a digital CCD camera (QImaging, Tucson, AZ, USA). Images were analyzed and the intensities were measured using ImageJ software (version 1.4.22, National Institutes of Health, Bethesda, MD, USA). 

### 4.5. Flow Cytometric Analysis of γH2AX

MDA-MB-231 cells were seeded in a 6-well plate at a concentration of 2 × 10^5^ cells/well. The cells were then incubated at 37 °C with 5% CO_2_ for 24 h. Next, **1** (1 μM), doxorubicin (1 μM), or cisplatin (10 μM) was then added and incubated for 24 h. The primary goal was to demonstrate that complex **1** can significantly higher levels of γH2AX compared to those treated with cisplatin or doxorubicin, even when a higher dosage of cisplatin was used. Through numerous pilot experiments with varying concentrations, we determined that the concentrations used in this study were optimal for obtaining measurable results. The live cells were collected, 250 μL BD permeabilization solution was added to resuspend the cells, and then the cells were incubated for 20 min at 4 °C. The cell pellets were then collected, washed twice with 1× BD Perm/Wash buffer, and resuspended in 50 µL of buffer. The samples were treated with Alexa 488-anti γH2AX antibody solution and incubated in the dark at room temperature for 60 min. The final cell pellets were resuspended in 200 μL of PBS containing 0.5% BSA. Then, they were analyzed with BD Accuri C6 flow cytometer using the FL-1 channel. The data were analyzed using FlowJo, version 10.8 (FlowJo LLC, Ashland, OR, USA).

### 4.6. Flow Cytometric Analysis of Apoptosis

MDA-MB-231 cells were seeded in a 6-well plate at a concentration of 2 × 10^5^ cells/well. Cells were then incubated at 37 °C, 5% CO_2_ for 72 h. Next, **1** (0.5 μM) was added and incubated for 72 h. Both viable and nonviable cells were collected, resuspended in 1 mL of phosphate-buffered saline (PBS), and quantified. Then, 1× binding buffer from an FITC Annexin V Apoptosis Detection Kit 1 (BD Biosciences, Franklin Lakes, NJ, USA) was added to achieve a concentration of 10^6^ cells/mL. The 100 µL cell solution was transferred to a fresh 2 mL Eppendorf tube, and both Annexin V-FITC and PI solutions were added to the cells. The cells were incubated for 15 min at room temperature in the absence of light and then diluted to a volume of 400 µL by adding the appropriate amount of binding 1× buffer. Cells were then analyzed with FL-1 and FL-3 channels on a BD Accuri C6 flow cytometer and the subsequent datasets were analyzed using FlowJo, version 10.8 (FlowJo LLC, Ashland, OR, USA).

## Data Availability

The original contributions presented in the study are included in the article/supplementary material, further inquiries can be directed to the corresponding author.

## Supplementary Materials

The following supporting information can be downloaded at: https://www.mdpi.com/article/10.3390/molecules29174095/s1, Figures S1–S6: Synthesis and characterization of the amphiphilic Pt(IV)–doxorubicin conjugate (**1**) and the representative killing curves from MTT assays.

## References

[1] Wang D., Lippard S.J. (2005). Cellular processing of platinum anticancer drugs. Nat. Rev. Drug Discov..

[2] Kelland L. (2007). The resurgence of platinum-based cancer chemotherapy. Nat. Rev. Cancer.

[3] Jamieson E.R., Lippard S.J. (1999). Structure, recognition, and processing of cisplatin-DNA adducts. Chem. Rev..

[4] Todd R.C., Lippard S.J. (2009). Inhibition of transcription by platinum antitumor compounds. Metallomics.

[5] Johnstone T.C., Suntharalingam K., Lippard S.J. (2016). The Next Generation of Platinum Drugs: Targeted Pt(II) Agents, Nanoparticle Delivery, and Pt(IV) Prodrugs. Chem. Rev..

[6] Bayat Mokhtari R., Homayouni T.S., Baluch N., Morgatskaya E., Kumar S., Das B., Yeger H. (2017). Combination therapy in combating cancer. Oncotarget.

[7] Kar A., Agarwal S., Singh A., Bajaj A., Dasgupta U. (2024). Insights into molecular mechanisms of chemotherapy resistance in cancer. Transl. Oncol..

[8] Li Y., Lin W. (2023). Platinum-based combination nanomedicines for cancer therapy. Curr. Opin. Chem. Biol..

[9] Zhong T., Yu J., Pan Y., Zhang N., Qi Y., Huang Y. (2023). Recent Advances of Platinum-Based Anticancer Complexes in Combinational Multimodal Therapy. Adv. Healthc. Mater..

[10] Jogadi W., Zheng Y.R. (2023). Supramolecular platinum complexes for cancer therapy. Curr. Opin. Chem. Biol..

[11] Zhang P., Zhou Z., Long W., Yan Y., Li Y., Fu T., Liu Y., Zhao Z., Tan W., Stang P.J. (2022). Self-assembled Pt(II) metallacycles enable precise cancer combination chemotherapy. Proc. Natl. Acad. Sci. USA.

[12] Chen J., Zhang Y., Meng Z., Guo L., Yuan X., Chai Y., Sessler J.L., Meng Q., Li C. (2020). Supramolecular combination chemotherapy: A pH-responsive co-encapsulation drug delivery system. Chem. Sci..

[13] Samanta S., Quigley J., Vinciguerra B., Briken V., Isaacs L. (2017). Cucurbit[7]uril Enables Multi-Stimuli-Responsive Release from the Self-Assembled Hydrophobic Phase of a Metal Organic Polyhedron. J. Am. Chem. Soc..

[14] Zhang G., Zhu Y., Wang Y., Wei D., Wu Y., Zheng L., Bai H., Xiao H., Zhang Z. (2019). pH/redox sensitive nanoparticles with platinum(iv) prodrugs and doxorubicin enhance chemotherapy in ovarian cancer. RSC Adv..

[15] Miller M., Askevold B., Mikula H., Kohler R., Pirovich D., Weissleder R. (2017). Nano-palladium is a cellular catalyst for in vivo chemistry. Nat. Commun..

[16] Samanta S.K., Moncelet D., Briken V., Isaacs L. (2016). Metal–Organic Polyhedron Capped with Cucurbit[8]uril Delivers Doxorubicin to Cancer Cells. J. Am. Chem. Soc..

[17] Hess U., Shahabi S., Treccani L., Streckbein P., Heiss C., Rezwan K. (2017). Co-delivery of cisplatin and doxorubicin from calcium phosphate beads/matrix scaffolds for osteosarcoma therapy. Mater. Sci. Eng. C Mater. Biol. Appl..

[18] Sreekanth V., Medatwal N., Pal S., Kumar S., Sengupta S., Bajaj A. (2017). Molecular Self-Assembly of Bile Acid-Phospholipids Controls the Delivery of Doxorubicin and Mice Survivability. Mol. Pharm..

[19] Jin Y., Wang Y., Liu X., Zhou J., Wang X., Feng H., Liu H. (2020). Synergistic Combination Chemotherapy of Lung Cancer: Cisplatin and Doxorubicin Conjugated Prodrug Loaded, Glutathione and pH Sensitive Nanocarriers. Drug Des. Dev. Ther..

[20] Xue X., Wu Y., Xu X., Xu B., Chen Z., Li T. (2021). pH and Reduction Dual-Responsive Bi-Drugs Conjugated Dextran Assemblies for Combination Chemotherapy and In Vitro Evaluation. Polymers.

[21] Zheng Y.-R., Suntharalingam K., Johnstone T.C., Yoo H., Lin W., Brooks J.G., Lippard S.J. (2014). Pt(IV) Prodrugs Designed to Bind Non-Covalently to Human Serum Albumin for Drug Delivery. J. Am. Chem. Soc..

[22] Jayawardhana A.M.D.S., Stilgenbauer M., Datta P., Qiu Z., Mckenzie S., Wang H., Bowers D., Kurokawa M., Zheng Y.R. (2020). Fatty acid-like Pt(IV) prodrugs overcome cisplatin resistance in ovarian cancer by harnessing CD36. Chem. Commun..

[23] Jayawardhana A.M.D.S., Zheng Y.R. (2022). Interactions between mitochondria-damaging platinum(IV) prodrugs and cytochrome c. Dalton Trans..

[24] Awuah S.G., Zheng Y.R., Bruno P.M., Hemann M.T., Lippard S.J. (2015). A Pt(IV) Pro-drug Preferentially Targets Indoleamine-2,3-dioxygenase, Providing Enhanced Ovarian Cancer Immuno-Chemotherapy. J. Am. Chem. Soc..

[25] Wei D., Yu Y., Zhang X., Wang Y., Chen H., Zhao Y., Wang F., Rong G., Wang W., Kang X. (2020). Breaking the Intracellular Redox Balance with Diselenium Nanoparticles for Maximizing Chemotherapy Efficacy on Patient-Derived Xenograft Models. ACS Nano.

[26] Jayawardhana A.M.D.S., Bhandari S., Kaspi-Kaneti A.W., Kshetri M., Qiu Z., Cheline M., Shen H., Dunietz B.D., Zheng Y.R. (2023). Visible light-activatable platinum(IV) prodrugs harnessing CD36 for ovarian cancer therapy. Dalton Trans..

[27] Feng W.W., Wilkins O., Bang S., Ung M., Li J., An J., Del Genio C., Canfield K., DiRenzo J., Wells W. (2019). CD36-Mediated Metabolic Rewiring of Breast Cancer Cells Promotes Resistance to HER2-Targeted Therapies. Cell Rep..

[28] Miller M., Zheng Y., Suresh G., Pfirschke C., Zope H., Engblom C., Kohler R., Iwamoto Y., Yang K., Askevold B. (2015). Tumour-associated macrophages act as a slow-release reservoir of nano-therapeutic Pt(IV) pro-drug. Nat. Commun..

[29] Zhou F., Feng B., Yu H., Wang D., Wang T., Ma Y., Wang S., Li Y. (2019). Tumor Microenvironment-Activatable Prodrug Vesicles for Nanoenabled Cancer Chemoimmunotherapy Combining Immunogenic Cell Death Induction and CD47 Blockade. Adv. Mater..

[30] Kang X., Wang Y., Chen Z., Wu Y., Chen H., Yang X., Yu C. (2020). Imidazole modified Pt(iv) prodrug-loaded multi-stage pH responsive nanoparticles to overcome cisplatin resistance. Chem. Commun..

[31] Ma J., Wang Q., Huang Z., Yang X., Nie Q., Hao W., Wang P.G., Wang X. (2017). Glycosylated Platinum(IV) Complexes as Substrates for Glucose Transporters (GLUTs) and Organic Cation Transporters (OCTs) Exhibited Cancer Targeting and Human Serum Albumin Binding Properties for Drug Delivery. J. Med. Chem..

[32] Abu Ammar A., Raveendran R., Gibson D., Nassar T., Benita S. (2016). A Lipophilic Pt(IV) Oxaliplatin Derivative Enhances Antitumor Activity. J. Med. Chem..

[33] Kshetri M., Jogadi W., Alqarni S., Datta P., Cheline M., Sharma A., Betters T., Broyles D., Zheng Y.R. (2023). Exploring the Impact of Head Group Modifications on the Anticancer Activities of Fatty-Acid-like Platinum(IV) Prodrugs: A Structure-Activity Relationship Study. Int. J. Mol. Sci..

[34] Sreekanth V., Bajaj A. (2019). Recent Advances in Engineering of Lipid Drug Conjugates for Cancer Therapy. ACS Biomater. Sci. Eng..

[35] Morstein J., Capecchi A., Hinnah K., Park B., Petit-Jacques J., Van Lehn R.C., Reymond J.L., Trauner D. (2022). Medium-Chain Lipid Conjugation Facilitates Cell-Permeability and Bioactivity. J. Am. Chem. Soc..

[36] Couvreur P., Lepetre-Mouelhi S., Garbayo E., Blanco-Prieto M.J. (2023). Self-assembled lipid–prodrug nanoparticles. Nat. Rev. Bioeng..

[37] Huang L., Yang J., Wang T., Gao J., Xu D. (2022). Engineering of small-molecule lipidic prodrugs as novel nanomedicines for enhanced drug delivery. J. Nanobiotechnol..

[38] Coppens E., Desmaële D., Mougin J., Tusseau-Nenez S., Couvreur P., Mura S. (2021). Gemcitabine Lipid Prodrugs: The Key Role of the Lipid Moiety on the Self-Assembly into Nanoparticles. Bioconjugate Chem..

[39] Irby D., Du C., Li F. (2017). Lipid–Drug Conjugate for Enhancing Drug Delivery. Mol. Pharm..

[40] Stilgenbauer M., Jayawardhana A.M.D.S., Datta P., Yue Z., Gray M., Nielsen F., Bowers D.J., Xiao H., Zheng Y.R. (2019). A spermine-conjugated lipophilic Pt(iv) prodrug designed to eliminate cancer stem cells in ovarian cancer. Chem. Commun..

