# Supplementary material for: Engineering Novel Amphiphilic Platinum(IV) Complexes to Co-Deliver Cisplatin and Doxorubicin
Source: Molecules. 2024 Aug 29;29(17):4095. doi: 10.3390/molecules29174095 (PMC11397443; doi:10.3390/molecules29174095)
Supplement: Supplementary file 1 [file molecules-29-04095-s001.zip › molecules-3135055-supplementary.pdf]

Engineering Novel Amphiphilic Platinum(IV) Complexes to Co-Deliver Cisplatin and Doxorubicin

Wjdan Jogadi [1], Man B. Kshetri [1], Suha Alqarni [1,2], Arpit Sharma [1], May Cheline [1], Md Al Amin [1], Cynthia Sheets [1], Angele Nsoure-Engohang [1], and Yao-Rong Zheng [1*]

1 Department of Chemistry and Biochemistry, Kent State University, 236 Integrated Sciences Building, Kent, OH 44242, USA
2 Department of Chemistry, University of Bisha, Bisha 67714, Saudi Arabia
* Correspondence: yzheng7@kent.edu; Tel.: +1-330-672-2267

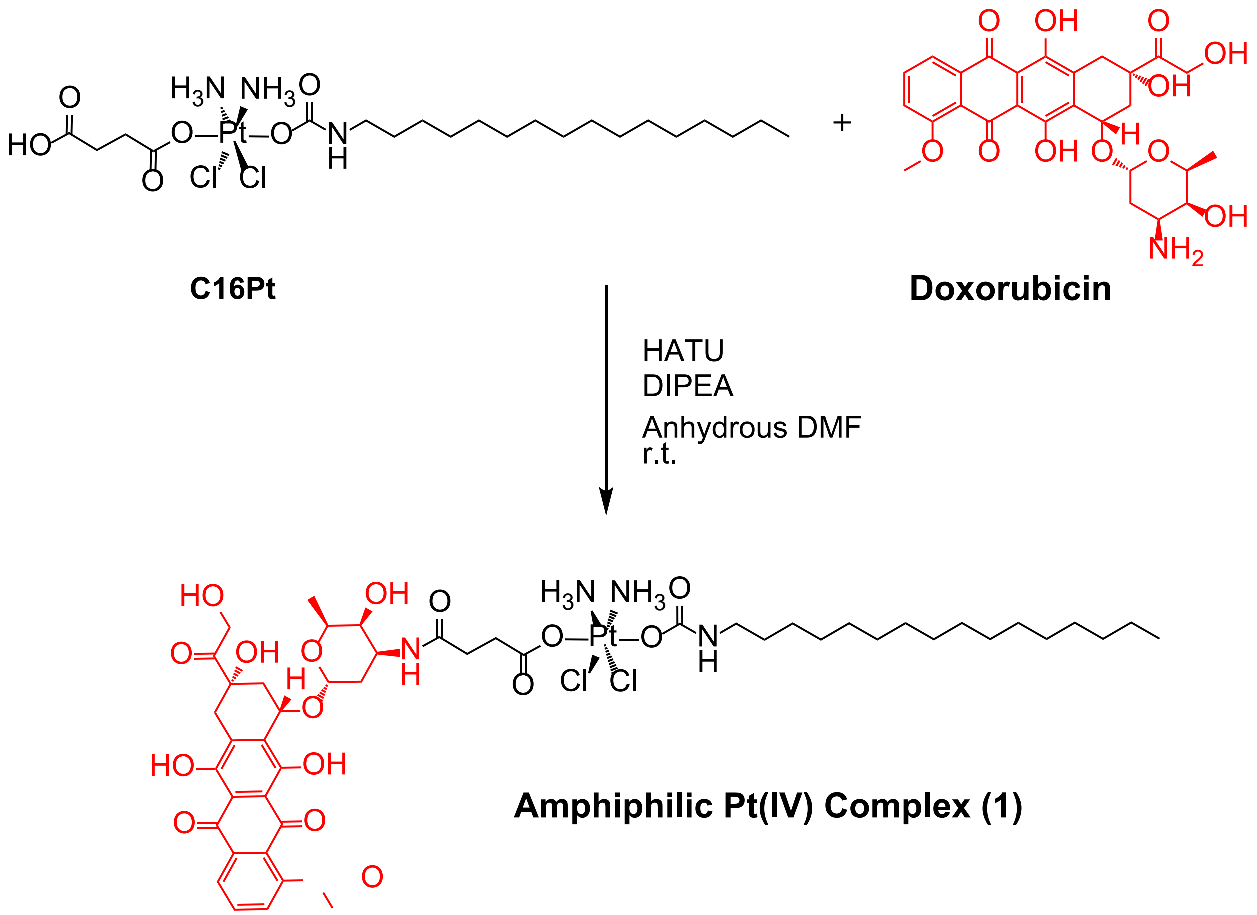

Figure S1. Synthesis of Amphiphilic Pt(IV)

Complex (**1**).

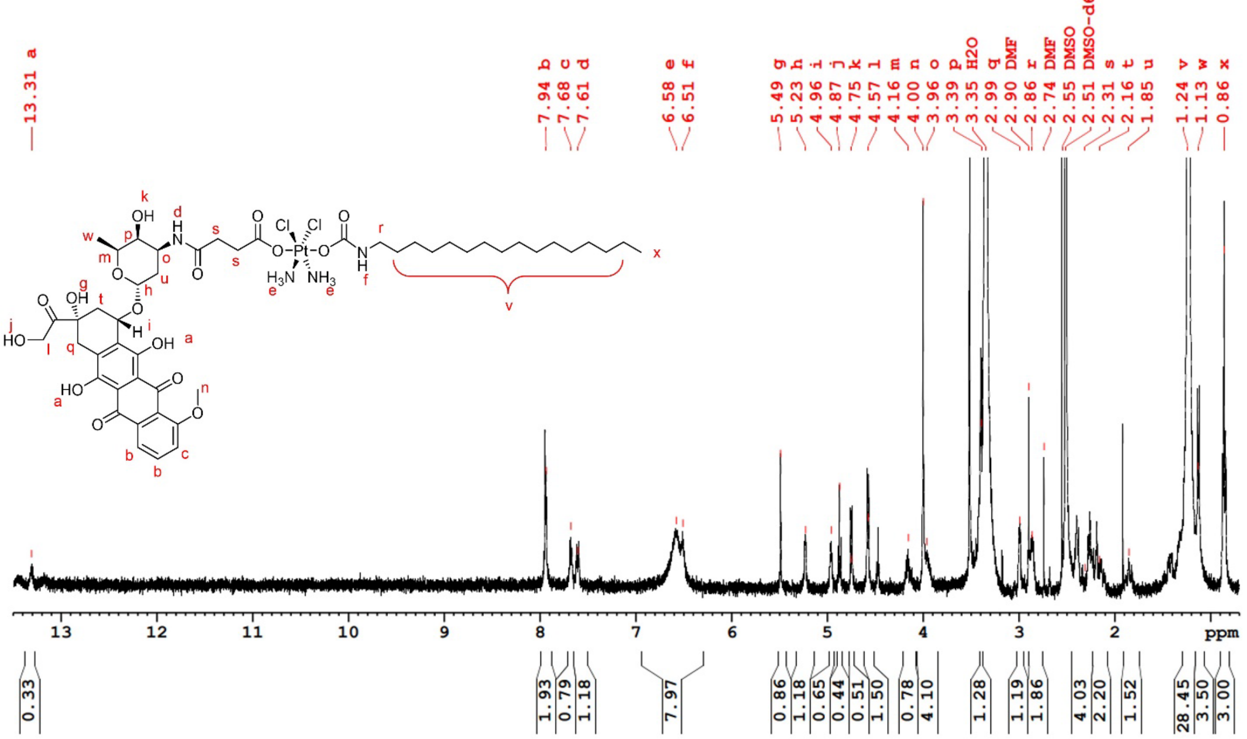

Figure S2. ^{1}H NMR spectrum of the amphiphilic Pt(IV)-doxorubicin conjugate (**1**) in DMSO-d_6.

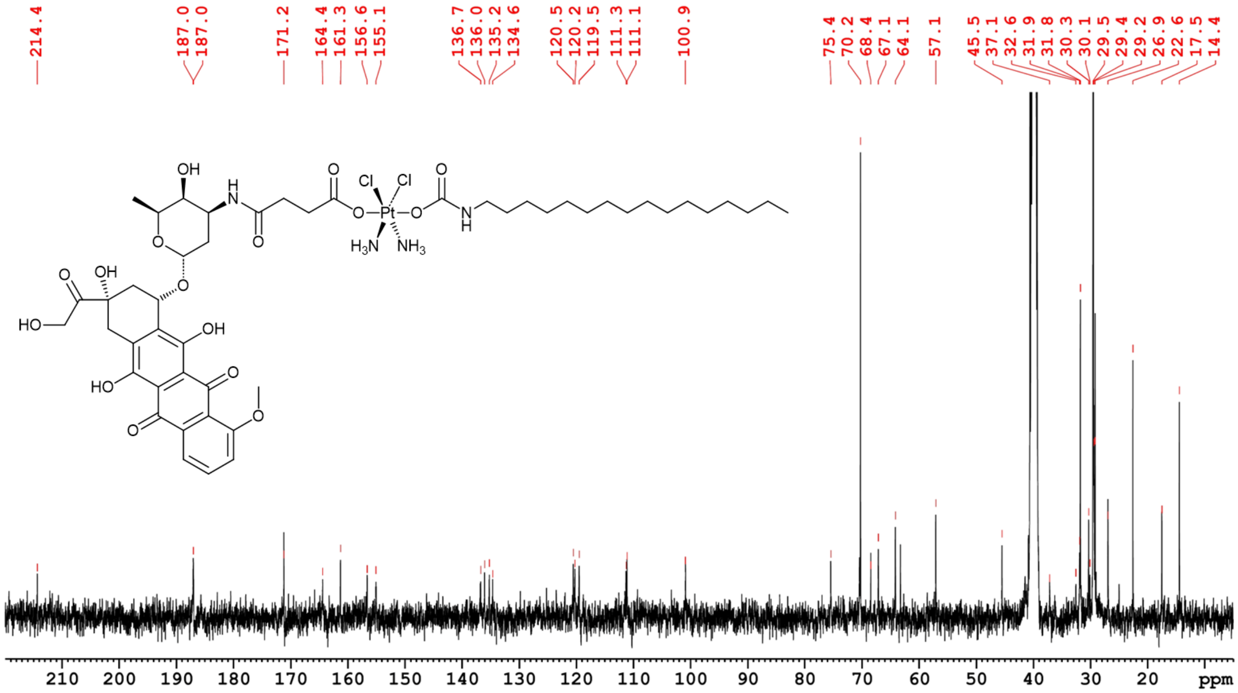

Figure S3. $^{13}C\{^{1}H\}$ NMR spectrum of the amphiphilic Pt(IV)-doxorubicin conjugate (**1**) in DMSO-d_6.

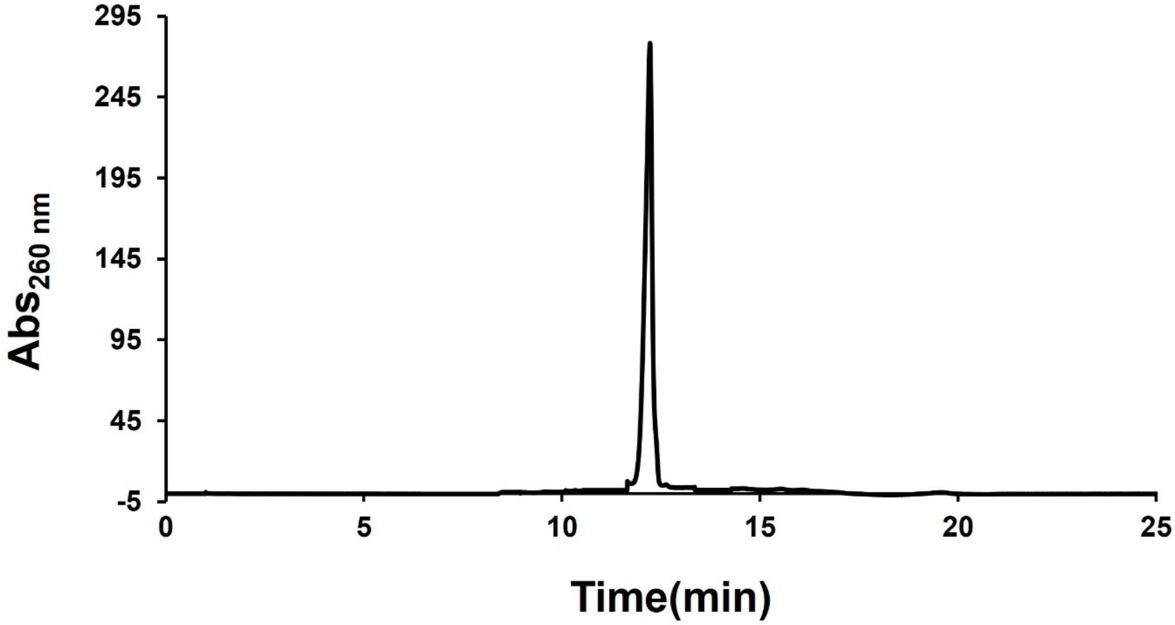

Figure S4. HPLC trace of the amphiphilic Pt(IV)-doxorubicin conjugate (**1**). Gradient: 0 min 5% B, 5 min 5% B, 10 min 85% B, 15 min 90% B, 20 min 98% B, 30 min 5% B (Solvent A is 0.1% TFA aqueous solution and B is acetonitrile).

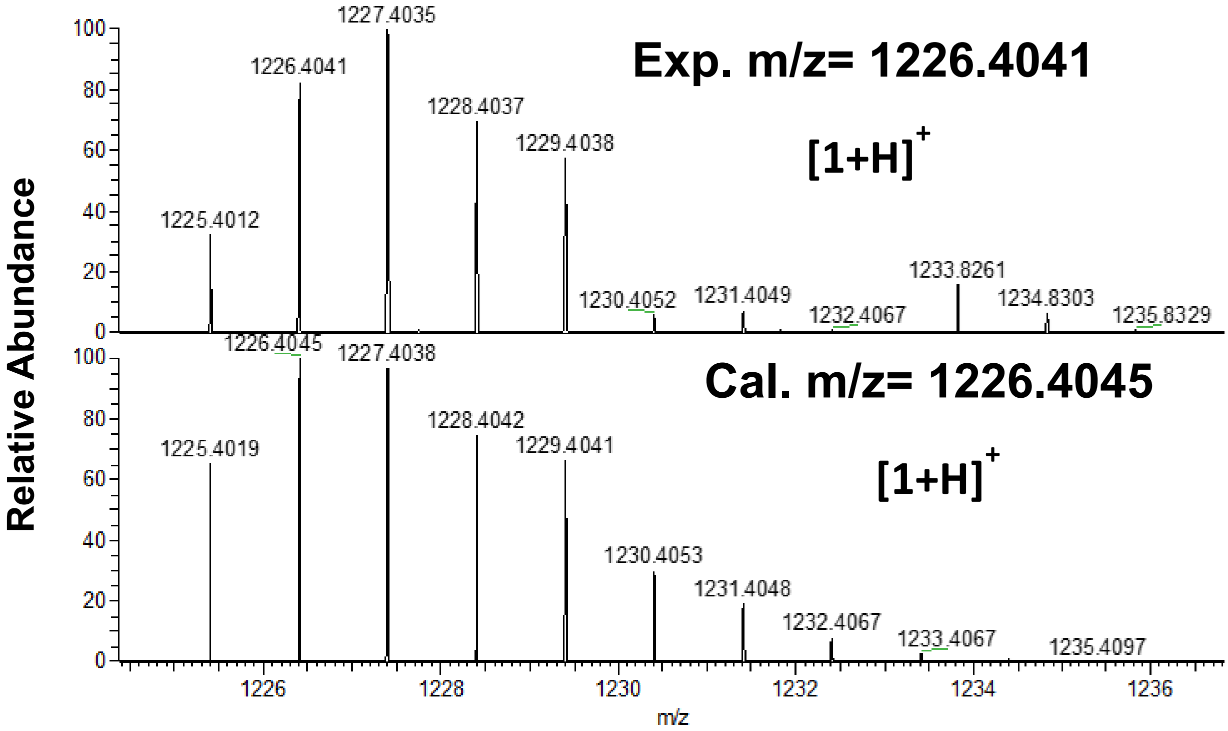

Figure S5. High resolution mass spectra of the amphiphilic Pt(IV)-doxorubicin conjugate (**1**).

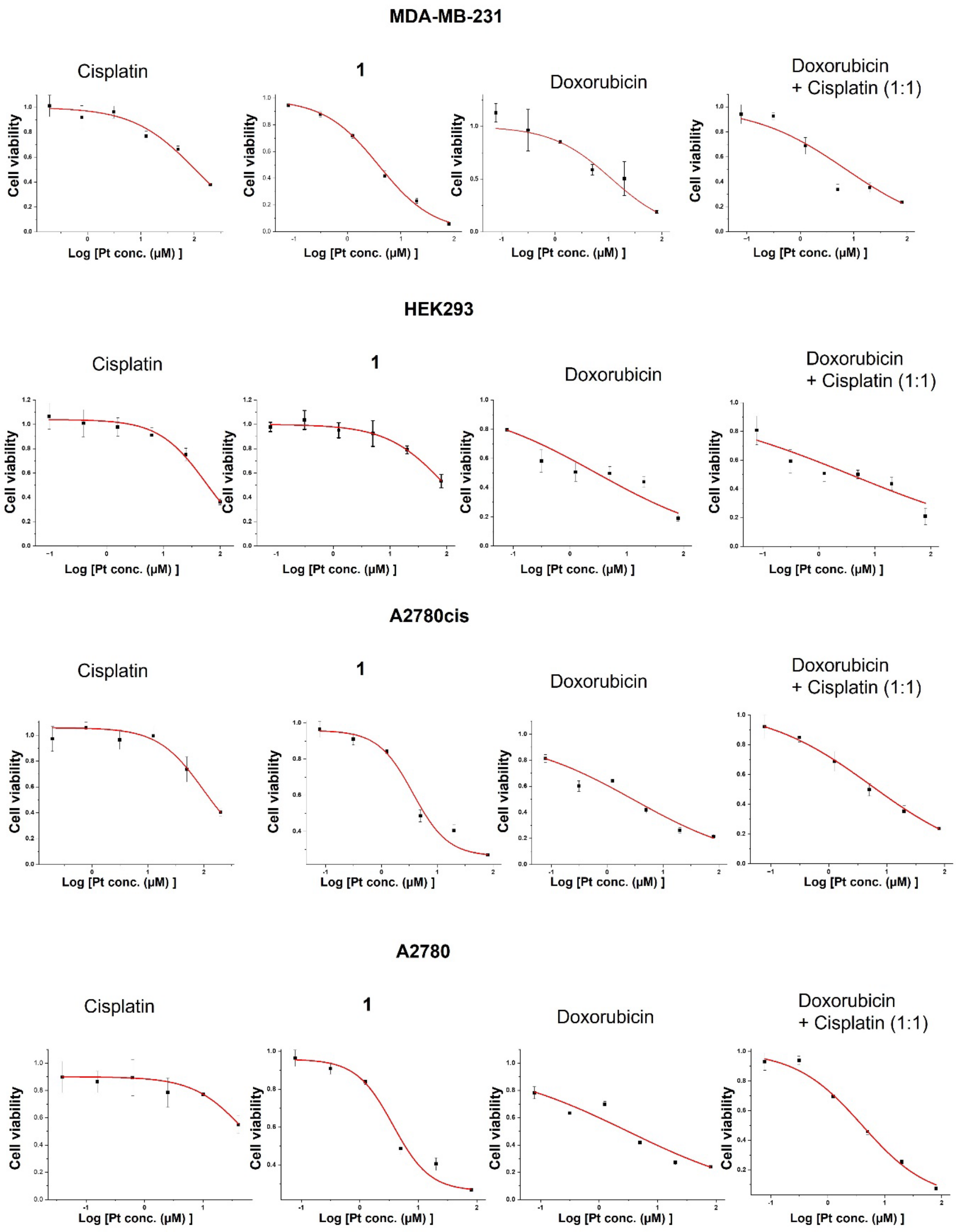

Figure S6. Representative killing curves from MTT assays.
